# The association between uric acid and erectile dysfunction in US adults: NHANES 2001–2004

**DOI:** 10.1186/s12882-024-03621-y

**Published:** 2024-06-03

**Authors:** Yi jun Wang, Ying he Chen, Lai lai Fan

**Affiliations:** Department of Urology, The Second Affiliated Hospital and Yuying Children’s Hospital of Wenzhou Medical University, Wenzhou, China

**Keywords:** Erectile dysfunction, Uric acid

## Abstract

**Background:**

—Recent evidence suggests that hyperuricemia may act as independent risk factors for erectile dysfunction (ED), in addition to the already established factors. The current evidence supporting this relationship remains insufficient.

**Methods and results:**

—A total of 3,810 participants from the NHANES pool between 2001 and 2004 were included in our study, comprising 1,093 individuals with ED and 2,717 individuals without ED. Univariable and multivariable logistic regression analyses were conducted to examine the relationship between uric acid (UA) and the prevalence of ED. In the fully adjusted model, no significant association was observed between UA and ED (OR = 1.02, 95% CI: 0.84–1.24), and no significant differences were noted among the various UA levels (*p* = 0.5). In our sensitivity analyses, employing a stricter definition for ED, no significant results were found in the fully adjusted model (OR = 0.85, 95% CI: 0.60–1.19). Furthermore, no significant differences were observed among the various UA levels (*p* = 0.083).

**Conclusions:**

—Our study did not establish a correlation between UA levels and ED. Nonetheless, further research with larger sample cohorts is required to verify these findings.

## Introduction

Erectile dysfunction (ED) is a commonly reported medical condition characterized by the chronic inability to achieve or maintain an erection of the penis [1]. Currently, increasing amount of evidence supports the vascular origin of ED. Hypertension, diabetes mellitus, hypercholesterolemia, smoking, obesity, and aging contribute to endothelial dysfunction, which can subsequently lead to ED [2]. Studies indicate that approximately 35% of men aged 60 and above experience ED, with this percentage increasing to around 50% among men over the age of 70 [3], and 86% of those aged over 80 years [4]. This occurrence rate places significant pressure on both partners and their overall quality of life.

Serum uric acid (UA) has been linked to endothelial dysfunction, oxidative stress, and inflammation and is increasingly recognized as a potential predictor of cardiovascular disease (CVD) risk [5]. Furthermore, the atherosclerotic CVD risk score has been demonstrated as a reliable tool to identify patients with ED in clinical practice [6]. Recent evidence suggests that hyperuricemia may act as independent risk factors for ED, in addition to the already established factors [7]. Inflammation may play an important role in both ED and hyperuricemia. The current evidence supporting this relationship remains insufficient.

However, high-quality designs for large samples were limited in previous studies. To further explore the relationship, we conducted a new study utilizing a large sample size and implementing comprehensive confounding adjustments.

## Methods

### Study design and population

The NHANES (National Health and Nutrition Examination Survey) is an ongoing nationwide survey conducted to assess the health and nutritional status of the general population in the United States. This is designed as a cross-sectional study that provides valuable insights into the health conditions and trends of Americans. The survey is conducted under the approval of the National Center for Health Statistics (NCHS) Ethics Review Board, ensuring adherence to ethical considerations (protocol number: 98 − 12). Informed consent is obtained from all participants who voluntarily participate in the survey. Detailed study protocols and findings are accessible on the Centers for Disease Control and Prevention (CDC) website in the United States (http://www.cdc.gov/nchs/nhanes.htm). This study was based on a sample of individuals who participated in two consecutive 2-year cycles of NHANES (2001–2002 and 2003–2004). This specific period was selected because information on ED was only available for the years 2001 to 2004.

**Fig. 1:**
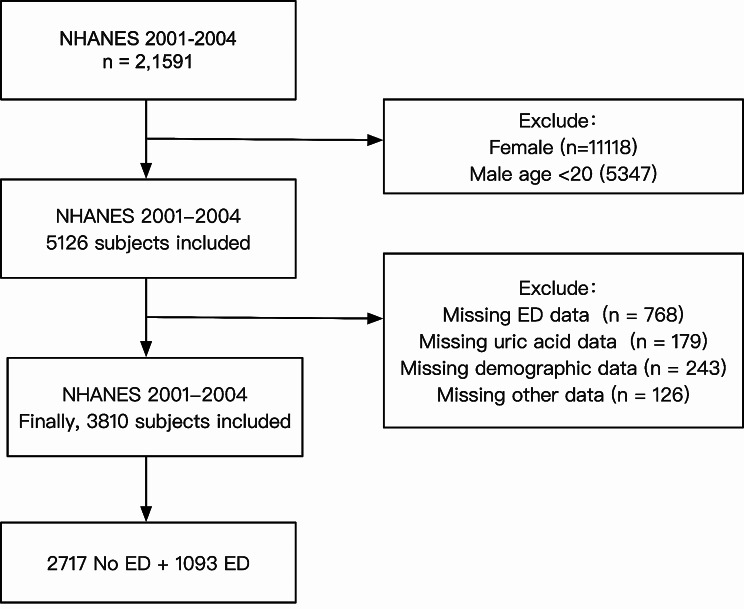
Flow chart of the sample selection process

A total of 21,591 participants from the NHANES pool between 2001 and 2004 were included as subjects in this study. Initially, women (*n* = 11,118) and men younger than 20 years of age (*n* = 5,347) were excluded. Further exclusions were based on the following criteria: (1) incomplete ED survey responses (*n* = 768); (2) unknown UA data (*n* = 179); (3) unknown demographic data, including age, race, education level, poverty income ratio (PIR), and marital status (*n* = 243); (4) unknown data regarding physical activity, body mass index (BMI), hypertension and diabetes (*n* = 126). Following the screening process, a final study cohort of 3,810 participants was included (Fig. 1).

### Assessment of ED

In this study, the interviews were conducted in a private room at the Mobile Examination Center (MEC) using an audio computer-assisted self-interview (ACASI) format. The assessment of ED as the outcome variable was carried out through a single question adapted from the Massachusetts Male Aging Study [8]. This self-reported ED item has demonstrated accurate prediction of clinician-diagnosed ED and may serve as a useful screening tool for referrals [9]. This question asked participants to describe their ability to obtain and maintain an erection sufficient for satisfactory intercourse. The response options for participants included “always or almost always able,” “usually able,” “sometimes able,” and “never able” when asked about their ability to maintain an erection. In this analysis, ED was defined as participants who responded as “sometimes able” or “never able” to maintain an erection. Conversely, participants who answered “always or almost always able” or “usually able” were classified as not having ED [10]. In a sensitivity analysis, we performed a stringent redefinition of ED by considering only those participants who responded “never able” to maintain an erection.

### Covariates of interest

This study considered various covariates, including age, race, PIR (a ratio of family income to poverty threshold), education level, BMI(Kg/m^2^), marital status, physical activity, hypertension, diabetes, CVD, hypercholesterolemia, arthritis, UA, serum creatinine, smoking status, and alcohol intake. Vigorous activities, defined as physical activities resulting in heavy sweating or substantial increases in breathing or heart rate, were distinguished from moderate activities, which involve engaging in activities for a minimum of 10 min that cause only light sweating or a slight to moderate increase in breathing or heart rate. Participants who reported having smoked at least 100 cigarettes throughout their lifetime were categorized as smokers. Among smokers, those indicating they currently smoke “every day” or “some days” were classified as current smokers, while those reporting not currently smoking at all were classified as former smokers. Nonsmokers were individuals who had smoked fewer than 100 cigarettes in their lifetime. Nondrinkers were participants who answered “no” to consuming at least 12 alcohol drinks in their entire life and in any one year. Former drinkers were participants who reported consuming at least 12 alcohol drinks during their life or in any one year but reported no alcohol consumption in the past 12 months. Current drinkers were study individuals who reported consuming at least 12 alcohol drinks during their life or in any one year and confirmed consuming at least one alcohol drink in the past 12 months [11]. CVD included participants who had previously been diagnosed with congestive heart failure, coronary heart disease, angina, heart attack, or stroke. Hypercholesterolemia was defined as participants with total cholesterol levels higher than 6.2 mmol/L [12]. In this study, participants were categorized into three groups based on their UA levels: <3.5 mg/dL, 3.5–7.0 mg/dL, and > 7.0 mg/dL [2]. A UA level higher than 7.0 mg/dL was considered indicative of hyperuricemia. The data on depression was insufficient for conducting the regression analysis.

### Statistical analysis

In this study, we accounted for the multistage design of the NHANES in all statistical analyses by systematically selecting sampling weights, strata, and primary sampling units. The baseline characteristics table presented unweighted medians and interquartile range (IQR) for continuous variables, and unweighted frequencies and percentages for categorical variables. To assess the differences between participants with or without ED in complex survey samples, we employed the Wilcoxon rank-sum test for continuous variables and the chi-squared test with Rao & Scott’s second-order correction for categorical variables. Weighted-univariable and weighted-multivariable logistic regression analyses were conducted to examine the relationship between UA and the prevalence of ED. Four models were used in the analyses of the association between UA and ED. In the sensitivity analysis, ED was redefined as participants who responded “never able” to maintain an erection.

A significance level of < 0.05 was used for two-sided p-values to determine statistical significance. All statistical analyses were conducted using R (version 4.3.1) from The R Foundation (http://www.R-project.org).

## Result

### Basic characteristics

Following the screening criteria shown in Fig. 1, a total of 3,810 eligible participants from NHANES 2001–2004 were included in the study. Among the participants, 1,093 individuals were identified as experiencing ED, while 2,717 individuals did not exhibit ED. An overview of the fundamental characteristics of participants in the United States was presented in Table 1. Significant statistical differences were observed between participants with ED and those without ED across various factors, including age, PIR, education level, BMI, marital status, physical activity, hypertension, diabetes, CVD, arthritis, serum creatinine, smoking status, and alcohol intake.

**Table 1:** Unweighted baseline characteristic of population included by a history of erectile dysfunction

			ED		
Characteristic	*N* ^1^	Overall, *N* = 3810 (100%)^2^	No, *N* = 2717 (80%)^2^	Yes, *N* = 1093 (20%)^2^	*P* Value^3^
**Age (years)**	3,810	44.0 (33.0, 55.0)	41.0 (31.0, 50.0)	63.0 (53.0, 73.0)	< 0.001
**Race**	3,810				0.13
Non-Hispanic White		2,116 (75%)	1,453 (74%)	663 (78%)	
Mexican American		767 (7.4%)	554 (7.7%)	213 (6.2%)	
Non-Hispanic Black		685 (9.2%)	530 (9.6%)	155 (7.7%)	
Other Hispanic		129 (4.4%)	89 (4.1%)	40 (5.5%)	
Other Race		113 (4.1%)	91 (4.3%)	22 (2.6%)	
**PIR**	3,810				< 0.001
> 1.3 and ≤ 3.5		1,463.0 (35.0%)	1,001.0 (33.5%)	462.0 (41.1%)	
> 3.5		1,429.0 (48.5%)	1,099.0 (50.6%)	330.0 (40.0%)	
≤ 1.3		918.0 (16.5%)	617.0 (15.9%)	301.0 (18.9%)	
**Education**	3,810				< 0.001
Above high school		1,813 (56%)	1,380 (59%)	433 (47%)	
High school or GED		918 (27%)	693 (27%)	225 (24%)	
Less than high school		1,079 (17%)	644 (14%)	435 (29%)	
**BMI**	3,810				< 0.001
< 25		1,090 (28%)	813 (30%)	277 (23%)	
> 30		1,101 (30%)	760 (28%)	341 (36%)	
≥ 25 and < 30		1,619 (42%)	1,144 (42%)	475 (41%)	
**Marital status**	3,810				< 0.001
Living alone		1,152 (29%)	881 (31%)	271 (22%)	
Married or living with partner		2,658 (71%)	1,836 (69%)	822 (78%)	
**Physical activity**	3,810				< 0.001
Low or unable		1,468 (32%)	923 (29%)	545 (45%)	
Moderate		1,060 (29%)	694 (27%)	366 (36%)	
Vigorous		1,282 (40%)	1,100 (44%)	182 (19%)	
**Hypertension**	3,810	1,230 (28%)	649 (22%)	581 (51%)	< 0.001
**Diabetes**	3,810	1,230 (28%)	649 (22%)	581 (51%)	< 0.001
**CVD**	3,790	529 (9.9%)	199 (5.6%)	330 (28%)	< 0.001
**Hypercholesterolemia**	3,808	572 (16%)	436 (17%)	136 (13%)	0.061
**Arthritis**	3,800	888 (20%)	441 (15%)	447 (42%)	< 0.001
**Alcohol intaking**	2,124				< 0.001
Current drinkers		1,497 (73%)	1,164 (76%)	333 (60%)	
Former drinkers		494 (20%)	300 (17%)	194 (33%)	
Nondrinkers		133 (6.3%)	91 (6.3%)	42 (6.4%)	
**Smoking**	2,713				< 0.001
Current smokers		1,024 (38%)	798 (38%)	226 (39%)	
Former smokers		176 (5.3%)	92 (3.8%)	84 (13%)	
Nonsmokers		1,513 (57%)	1,192 (58%)	321 (49%)	
**Uric acid**	3,810	6.00 (5.20, 6.90)	6.00 (5.20, 6.90)	6.00 (5.20, 7.00)	0.7
**Serum creatinine**	3,810	1.00 (0.90, 1.10)	1.00 (0.90, 1.10)	1.00 (0.90, 1.20)	< 0.001

PIR: poverty income ratio; BMI: body mass index; CVD: cardiovascular disease

### Relationship between UA and ED

The results of the survey-weighted logistic regression analyses are presented in Table 2. In the unadjusted model, no significant correlation was observed between UA and ED (OR = 1.04, 95% CI: 0.95–1.15), and no significant difference was found among the different UA levels (*P* = 0.11). After adjusting for age, race, PIR, education level, and BMI (model 2), the association between UA and ED remained non-significant (OR = 1.04, 95% CI: 0.93–1.16), and no significant difference was observed among the different UA levels (*P* = 0.4). Similar results were obtained in model 3, which additionally adjusted for hypertension, diabetes, CVD, arthritis, physical activity, marital status, hypercholesterolemia, and serum creatinine (OR = 1.00, 95% CI: 0.89–1.12). In model 4, further adjustment for smoking status and alcohol intake did not reveal a significant relationship (OR = 1.02, 95% CI: 0.84–1.24), and no significant difference was detected among the different UA levels (*P* = 0.5).

**Table 2:** Weighted-multivariable logistic regression for the association between uric acid and erectile dysfunction

Characteristic	Model 1			Model 2			Model 3			Model 4		
	OR^1^	95% CI^1^	*p*-value	OR^1^	95% CI^1^	*p*-value	OR^1^	95% CI^1^	*p*-value	OR^1^	95% CI^1^	*p*-value
**Uric acid**	1.04	0.95, 1.15	0.4	1.04	0.93, 1.16	0.5	1.00	0.89, 1.12	> 0.9	1.02	0.84, 1.24	0.8
**Uric acid levels**			0.11			0.4			0.4			0.5
< 3.5	1.00			1.00			1.00			1.00		
≥ 3.5 and ≤ 7.0	0.68	0.36, 1.31		0.74	0.32, 1.67		0.65	0.26, 1.58		0.40	0.05, 3.24	
> 7.0	0.86	0.44, 1.70		0.88	0.38, 2.05		0.73	0.29, 1.84		0.45	0.08, 2.70	

1OR = Odds Ratio, CI = Confidence Interval

Model 1: Unadjusted

Model 2: Adjusted for age, race, poverty income ratio, education level and body mass index

Model 3: Adjusted for model 2 covariates and for hypertension, diabetes, cardiovascular disease, arthritis, physical activity, marital status, hypercholesterolemia, creatinine

Model 4: Adjusted for model 3 covariates and for smoking status and alcohol intake

To minimize potential confounding factors and improve the accuracy of our findings, additional sensitivity analyses were conducted. This involved a rigorous redefinition of ED, specifically considering only those participants who responded “never able” to maintain an erection (Table 3). In the sensitivity analyses, which included 443 patients with ED and 3,367 patients without ED, no significant results were found in the fully adjusted model (OR = 0.85, 95% CI: 0.60–1.19), and no significant difference was observed among the different UA levels (*P* = 0.083).

**Table 3:** Weighted-multivariable logistic regression for the sensitivity analysis between uric acid and erectile dysfunction

Characteristic	Model 1			Model 2			Model 3			Model 4		
	OR^1^	95% CI^1^	*p*-value	OR^1^	95% CI^1^	*p*-value	OR^1^	95% CI^1^	*p*-value	OR^1^	95% CI^1^	*p*-value
**Uric acid**	1.04	0.93, 1.16	0.5	1.00	0.87, 1.15	> 0.9	0.95	0.82, 1.10	0.4	0.85	0.60, 1.19	0.3
**Uric acid levels**			**0.050**			0.4			0.3			0.083
< 3.5	1.00			1.00			1.00			1.00		
≥ 3.5 and ≤ 7.0	0.47	0.22, 1.01		0.45	0.11, 1.86		0.38	0.08, 1.72		0.15	0.02, 1.24	
> 7.0	0.62	0.29, 1.30		0.54	0.13, 2.19		0.41	0.09, 1.86		0.20	0.03, 1.55	

1OR = Odds Ratio, CI = Confidence Interval

Model 1: Unadjusted

Model 2: Adjusted for age, race, poverty income ratio, education level and body mass index

Model 3: Adjusted for model 2 covariates and for hypertension, diabetes, cardiovascular disease, arthritis, physical activity, marital status, hypercholesterolemia, creatinine

Model 4: Adjusted for model 3 covariates and for smoking status

## Discussion

Current research on the association between UA and ED primarily focuses on the relationship between hyperuricemia and ED [13–15], as well as gout and ED [16–18]. Gout is characterized by disruptions in UA metabolism, leading to recurrent episodes of arthritis pain and inflammation. Although hyperuricemia is a significant risk factor for developing gout, not every individuals with hyperuricemia will develop this condition. Hyperuricemia is also associated with an increased risk of several other health issues, including hypertension, CVD, metabolic syndrome, diabetes, and obesity [5].

Most studies available suggest that gout is an independent risk factor for ED. In the study conducted by Chung et al. [16], involving 19,368 gout patients, the findings indicated a 1.21-fold adjusted hazard ratio (HR) for the subsequent development of ED compared to the non-gout cohort (95% CI: 1.03–1.44). Similarly, the study by Roddy et al. [17], which included 9,653 gout patients, demonstrated a 1.31-fold adjusted HR (95% CI: 1.24–1.40) for the development of ED compared to individuals without gout. Furthermore, the study by Schlesinger et al. [18], involving 38,438 gout patients, revealed a 1.15-fold adjusted HR for the subsequent development of ED compared to the non-gout cohort (95% CI: 1.09–1.21). These studies collectively provide evidence supporting an increased risk of developing ED in individuals with gout compared to those without gout. However, none of these studies conducted subgroup analyses on UA. While the pathological relationship between them has not been confirmed, ED is a multifaceted condition involving various components of the erectile response, including organic, relational, and psychological factors. Among the organic causes of ED, vasculogenic ED is the most prevalent etiology [19]. Hypertension, diabetes, dyslipidemia, and smoking can lead to atherosclerosis and compound the vascular injury that increases the risk of ED [20–22]. Proponents emphasizing the pathogenic mechanisms of gout suggest that the inflammatory response associated with gout may contribute to vascular effects, potentially leading to ED. UA is not only associated with endothelial dysfunction but also with oxidative stress and inflammation, which adds complexity to the relationship between gout and ED [23].

The relationship between hyperuricemia and ED remains controversial. In our study, we did not find a significant association between UA levels and ED, nor did we observe a notable risk of ED in patients with hyperuricemia. To ensure the accuracy of our analysis, we employed stricter criteria for defining ED. Among individuals with hyperuricemia, no significant relationship was identified. It is worth noting that there are some contrasting findings in the literature. For instance, a cross-sectional study by Kanbay et al. [15], which included 149 ED patients, did not find an association between elevated UA levels and ED in their multivariate analysis. Conversely, another study by Kanbay et al. [10], involving 110 ED patients, observed that UA is an independent determinant of ED (OR: 1.76, 95% CI: 1.28–2.41). Additionally, a study by Pourmand et al. [11], with 251 ED patients, found that serum UA can serve as a predictive risk factor for ED (OR: 2.07, 95% CI: 1.63–2.64). A cross-sectional study by Gao et al. [24] with 513 ED patients found that UA might have a protective role in the development of ED through its relationship with the free androgen index. However, it is important to note that the scale of these studies are relatively small and may lack complete adjustment for confounding factors, thereby providing limited evidence. Given the conflicting findings and limitations of existing studies, further research with larger sample sizes and comprehensive adjustment for confounders is necessary to better understand the potential relationship between hyperuricemia and ED.

This study has certain limitations. Firstly, being a cross-sectional study, it does not establish causal relationships. Secondly, relying solely on the question “Have you ever had ED?” may not provide a comprehensive assessment of erectile function, and it does not allow for differentiation between different types of ED. Thirdly, the data on depression and specific medication intake are not available for analysis. Therefore, it is necessary to conduct more thoroughly designed prospective and multicenter studies to address these limitations.

## Conclusion

Although hyperuricemia is closely associated with gout, individuals with hyperuricemia may not develop gout in their lifetime. Existing studies have widely reported an association between gout and ED; however, our study did not find a correlation between UA levels and ED. Further research with large sample cohorts is necessary to confirm these findings.

## Data Availability

Detailed study protocols and findings can be accessed on the Centers for Disease. Control and Prevention website in the United States (http://www.cdc.gov/nchs/nhanes.htm).
